# The Effects of Duodenojejunal Omega Switch in Combination with High-Fat Diet and Control Diet on Incretins, Body Weight, and Glucose Tolerance in Sprague-Dawley Rats

**DOI:** 10.1007/s11695-017-2883-3

**Published:** 2017-08-24

**Authors:** Dominika Stygar, Tomasz Sawczyn, Bronisława Skrzep-Poloczek, Aleksander J. Owczarek, Natalia Matysiak, Marek Michalski, Łukasz Mielańczyk, Barbara Bażanów, Paweł Ziora, Piotr Choręza, Bogdan Doleżych, Konrad Wojciech Karcz

**Affiliations:** 1 Department of Physiology, School of Medicine with Dentistry Division in Zabrze, Medical University of Silesia, Katowice, Poland; 2 Department of Instrumental Analysis, School of Pharmacy with the Division of Laboratory Medicine in Sosnowiec, Medical University of Silesia, Katowice, Poland; 3 Department of Histology and Embryology, School of Medicine with the Division of Dentistry, Medical University of Silesia, Zabrze, Poland; 4 Department of Pathology, Faculty of Veterinary Medicine, Wrocław University of Environmental and Life Sciences, Wrocław, Poland; 5 Department of Animal Physiology and Ecotoxicology, Faculty of Biology and Environmental Protection, University of Silesia, Katowice, Poland; 6 Clinic of General, Visceral, Transplantation and Vascular Surgery, Hospital of the Ludwig Maximilian University, Munich, Germany

**Keywords:** Bariatric surgery, DJOS surgery, Obesity, Experimental rat model, Incretins, OGTT, Glucose tolerance, Insulin intolerance, GIP, GLP-1

## Abstract

**Background:**

Despite excellent results of bariatric surgery in the treatment of type 2 diabetes and weight loss in human subjects, some patients do not obtain desired results. One of the reasons for this is that not all patients follow caloric intake recommendations.

**Aim:**

The aim of this study was to investigate the effect of duodenojejunal omega switch (DJOS) surgery on body weight, glucose tolerance, and incretins in rats.

**Methods:**

DJOS and SHAM surgery were performed on rats maintained for 8 weeks on high-fat diet (HF) and control diet (CD), respectively. After surgery, four groups were kept on the same diet as before the surgery, and four groups had a changed diet (CD vs. HF and HF vs. CD) for the next 8 weeks. Glucose tolerance, insulin tolerance, glucose-stimulated insulin, glucagon-like peptide-1 (GLP-1) and gastric inhibitory polypeptide/glucose-dependent insulinotropic polypeptide (GIP) secretion, food intake, and body weight were measured.

**Results:**

A change of diet after surgery resulted in reduced glucose tolerance. Plasma insulin levels were lowered between DJOS and SHAM surgeries for the HF/HF and CD/HF groups. DJOS surgery did not reduce body weight in the studied groups, irrespective of diet. In the HF/HF group, ΔGLP-1 was lower for DJOS surgery in comparison with other groups. Differences of weight changes were observed for groups HF/HF and HF/CD. After DJOS surgery, ΔGIP was lower in the CD/HF group compared with HF/HF.

**Conclusions:**

Our results show that applications of different types of diets, before and after surgery, is a sensitive method for studies of mechanism of glucose intolerance after DJOS surgery.

## Introduction

One of the most effective treatments for long-term weight loss and energy control is bariatric surgery, where still new surgical techniques are being developed [1, 2]. Despite excellent results in the treatment of type 2 diabetes, and long-term weight loss in human subjects after bariatric surgery [3, 4], some patients that have undergone surgery do not achieve the expected positive results. It so happened that one of the reasons for this failure is that not all patients that undergo surgery follow caloric and macronutrient intake recommendations following the operation [5, 6]. Other studies show that significant reduction in dietary fat preference and intake contributed to reduced weight loss maintenance in rats and humans following the operation [7]. According to Chikunguwo et al. [8], 40% of patients who underwent metabolic surgery showed a recurrence of T2DM and an increase in body weight. The reduction of T2DM and decreased obesity are connected with stable weight loss, age, gender, sex, and BMI before and after surgery [9–12]. In experimental animal models, such as the one involving Sprague-Dawley rats, diet-induced obesity and increases in insulin and lipid levels were observed but not always with an associated increase in blood glucose levels [8, 13, 14]. In the spontaneously diabetic rat model, Zucker diabetic fatty (ZDF), a high-fat diet (HF) accelerated the onset and severity of hyperglycemia [15]. It has also been reported that variations of metabolic response to diet-induced obesity for different animal strains are likely to be related to their genetic backgrounds. [16]. Glucagon-like peptide-1 (GLP-1) and gastric inhibitory polypeptide/glucose-dependent insulinotropic polypeptide (GIP) are incretins and peptide hormones that are released from the gastrointestinal tract into the circulatory system in response to nutrient ingestion that ameliorate glucose-stimulated insulin secretion. GLP-1 is known to stimulate insulin release from pancreatic β-cells and inhibit secretion of glucagon from α-cells. GLP-1 further improves peripheral insulin sensitivity and promotes β-cell proliferation. GIP is secreted from K cell granules concentrated in the upper small intestine, and GLP-1 is secreted from L cell granules, located in the lower small intestine and colon [17–19]. It is known that a diet rich in nutrients, as well as bariatric surgery, are both strong stimulants of GLP-1 and GIP secretion [18–20].

With these data in mind, our aim was to study the effect of duodenojejunal omega switch (DJOS) surgery on glucose administration parameters. In our experimental design, we simulated the observations that not all patients reduce dietary fat intake after surgery by maintaining some animals on a HF after surgery. We also included the possibility that after surgery patients may switch from a regular diet to a HF and from a HF to a regular diet. Then we assessed the effect of duodenojejunal omega switch surgery in combination with CD, and a HF diet, before and after surgery, on body weight, glucose tolerance, and insulin resistance.

## Materials and Methods

### Animals and Diets

Male Sprague-Dawley rats (Charles River Breeding Laboratories, Wilmington, MA) aged 7 weeks, 200 ± 7 g, were housed at a 12-h light–dark cycle, 22 °C, and 40–60% humidity. All rats had free access to water and rat food (ProvimiKliba AG, Kaiseraugst, Switzerland). The control group was maintained on ssniff® EF R/M. Obesity was induced by placing the animals on a HF (23.0 kJ/g, 59% fat, 27% carbohydrate, and 14% (EF RAT [E15744] Ssniff Spezialdiäten GmbH) for an average of 2 months. Animals maintained on the HF diet were pair-fed (kcals) with the animals exposed to an ad libitum control diet. The energy content of the high-fat and standard diets were 5.04 and 3.59 kcal/g (20.1 and 15.0 kJ/g), respectively. Since there were multiple rats in each cage, the calorie intake was calculated by dividing the total cage calorie intake by the number of animals per cage. Rats were fasted overnight before surgery and given oral glucose tolerance tests (OGTTs).

### Experimental Design

**Fig. 1:**
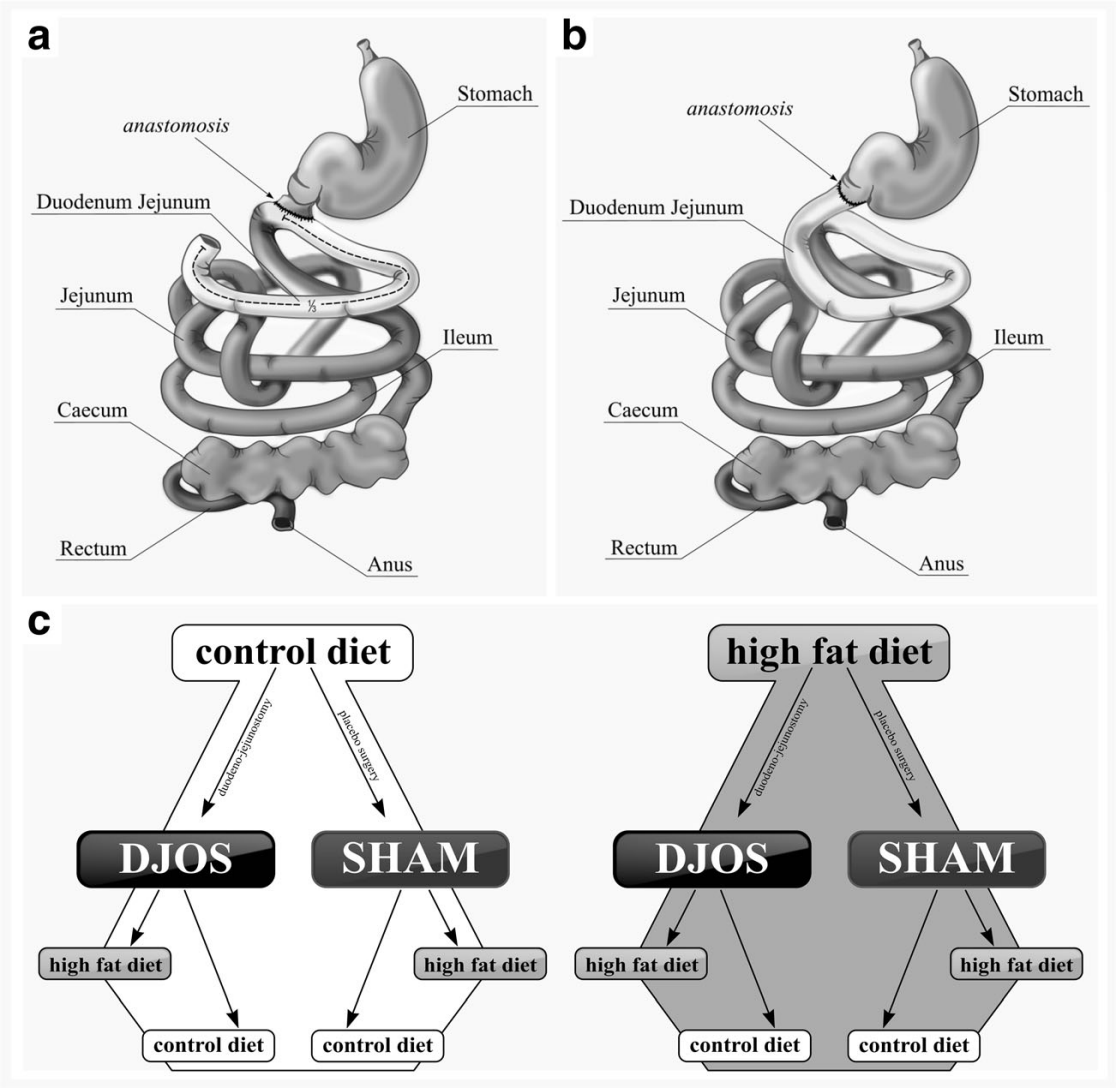
**a** Schematic illustration of DJOS and **b** SHAM surgery, respectively. **c** Scheme of experimental groups

After 1 week of acclimatization, the rats were assigned to the CD (*n* = 28) and HF groups (*n* = 28). After 8 weeks, both groups underwent SHAM (*n* = 14) and DJOS (*n* = 14) surgeries (Fig. 1b, c). After the surgery, animals from the CD and HF groups were divided further into eight groups: seven animals after surgery were kept on the same diet as before the surgery, and another seven from the group had a changed diet (Fig. 1a). Number of rats was kept as small as possible in consideration of the “3Rs” for the humane treatment of animals [21]. DJOS and SHAM surgeries were performed when the rats’ average body weight was 480 ± 50 g. The numbers of rats that survived in the experimental groups was seven; in the HF/SHAM/CD group, the number of rats that survived was only six. Anesthesia was administered according to previously described methodology for applying analgesia and antibiotic prophylaxis [15, 20]. A DJOS was performed according to Karcz et al. methodology [2] (Fig. 1b, c). Before performing the duodenoenterostomy, the length of the small bowel was measured to account for inter-individual differences (86.1 ± 6.47 cm). Briefly, the gastric volume was left intact, whereas the entire duodenum and the proximal jejunum were bypassed. The stomach was separated from the duodenum at the point just below the pylorus. The distal part of the transected duodenum was closed using Prolene 6/0 (Ethicon). The position of the duodenoenterostomy was determined to be at the aboral of the Treitz ligament, located approximately at one third of the total small bowel length for DJOS. The duodenojejunostomy was performed as a simple antecolic, continuous end-to-side hand-sewn extramucosal anastomosis using 6–0 sutures [2]. For the SHAM operation, transections and re-anastomosis of the gastrointestinal tract were performed at the corresponding sites where enterotomies were performed for the duodenojejunostomy, thereby maintaining the physiological conduit of food passage through the bowel. Weight, food intake, fasting, and non-fasting blood glucose were tracked for 8 weeks after surgery for all groups.

### OGTT, Blood, and Tissue Collection

A total of ten OGTTs were performed to assess glucose metabolism and were performed at 8 weeks before surgery and 8 weeks after surgery. For OGTTs, anesthesia was induced and maintained using isoflurane 2% and oxygen flow at 2 L/min breathing rate. The OGTT was initiated after the placement of an orogastric tube (central venous catheter, Arrow International Inc., Reading, Penn) infusing a 40% glucose solution at a dosage of 1.5 g/kg. The amount of glucose was determined via tail snip at 0, 10, 30, 60, 90, and 120 min using a Glucometer (Ascensia Elite, Bayer Corp, Monheim, Germany). The first and last OGTTs included blood collection from the right tail vein for hormone analysis. The right tail vein was cannulated using a 26-gauge cannula. Blood was drawn at 0 and 30 min after oral glucose was given using tubes containing 10 μl EDTA (Sigma-Aldrich, St. Louis, USA) and 4 μl DPP-4 inhibitor (DRG Instruments GmbH, Marburg, Germany). At 8 weeks after surgery, blood for additional hormone analysis was collected from the abdominal aorta via tubes containing 10 μl EDTA (Sigma-Aldrich, St. Louis, MO, USA). After centrifugation at 4000 rpm for 10 min at 4 °C, plasma samples were collected and snap frozen in liquid nitrogen and stored at −80 °C until analysis. After blood sampling, tissues were harvested and the animals were euthanized. White adipose was explanted and snap frozen in liquid nitrogen and stored at −80 °C until further analysis. All experimental procedures were approved by the Ethical Committee for Animal Experimentation of the Medical University of Silesia (58/2014). All applicable institutional and/or national guidelines for the care and use of animals were followed (directive 2010/63/EU).

### Hormonal Assessment

For hormonal evaluation, blood samples were collected from the right tail vein, which was dissected and cannulated using a 26-gauge cannula. Blood was drawn via the cannula at 0 and 30 min after oral glucose was given, using tubes containing 10 μl EDTA (Sigma-Aldrich, St. Louis, USA) and 4 μl DPP-4 inhibitor (DRG Instruments GmbH, Marburg, Germany). After centrifugation at 4000 rpm for 10 min at 4 °C, samples were snap frozen in liquid nitrogen and stored at −80 °C until analysis. Insulin, GLP-1 (7-36) and glucose-dependent insulinotropic peptide, GIP serum concentrations were assessed in duplicates by sandwich ELISA kits (Cloud-Clone Corp., USA).

### HOMA-IR

A homeostatic model assessment of insulin resistance (HOMA-IR) was calculated according to Matthews et al. [22].

### Liver Histology

#### Transmission Electron Microscopy Analysis

Immediately after collection, samples of liver were fixed in 2.5% glutaraldehyde (SERVA Electrophoresis GmbH-Heidelberg, Germany) in cacodylate buffer (pH 7.4) for 2 h at room temperature and then washed several times in the same buffer. Subsequently, the tissue was post-fixed in 1% osmium tetroxide (Polysciences Inc., Niles, Illinois, USA), dehydrated in a graded series of ethanol (50, 70, 90, and 96%) and propylene oxide. The samples were then infiltrated in 2:1 (vol:vol) and 1:2 (vol:vol) propylene oxide/Epon 812 mixtures, embedded in Epon 812 epoxy resin (SERVA Electrophoresis GmbH-Heidelberg, Germany), then polymerized for 48 h at 60 °C. Ultrathin sections were cut from representative samples with a diamond knife (45; RMC, Tucson, USA) using a Reichert OmU-3 ultramicrotome (Reichert, Vienna, Austria), mounted on 300-mesh copper grids and stained with 0.5% aqueous uranyl acetate and lead citrate (LAURYLAB Saint-Fons, France) using a Leica EM AC 20 stainer (Leica Microsystems, Vienna, Austria). After air drying of the grids, they were examined in a TECNAI™ G2 12 Spirit BioTWIN transmission electron microscope (FEI, Eindhoven, The Netherlands) at 120 kV. Images from representative regions were captured with a Morada CCD camera (Olympus Soft Imaging System Solutions GMBH, Münster, Germany).

### Statistical Analysis

Statistical analysis was performed using STATISTICA 12 PL (StatSoft, Inc. (2014). STATISTICA (data analysis software system), version 12. www.statsoft.com), StataSE 12.0 (StataCorp LP, TX, USA). Statistical significance was set at a *p* < 0.05. All tests were two-tailed. Interval data were expressed as mean value ± standard deviation in the case of normal distribution or as median/inter-quartile range in the case of data with skewed or non-normal distribution. The distribution of variables was evaluated by the Shapiro-Wilk test, and the homogeneity of variances was assessed by the Levene test. For comparison of data, the two-way ANOVA for inter-group comparisons and repeated measurement analysis were used with post hoc contrast analysis.

## Results

### OGGT Time Profiles

**Fig. 2:**
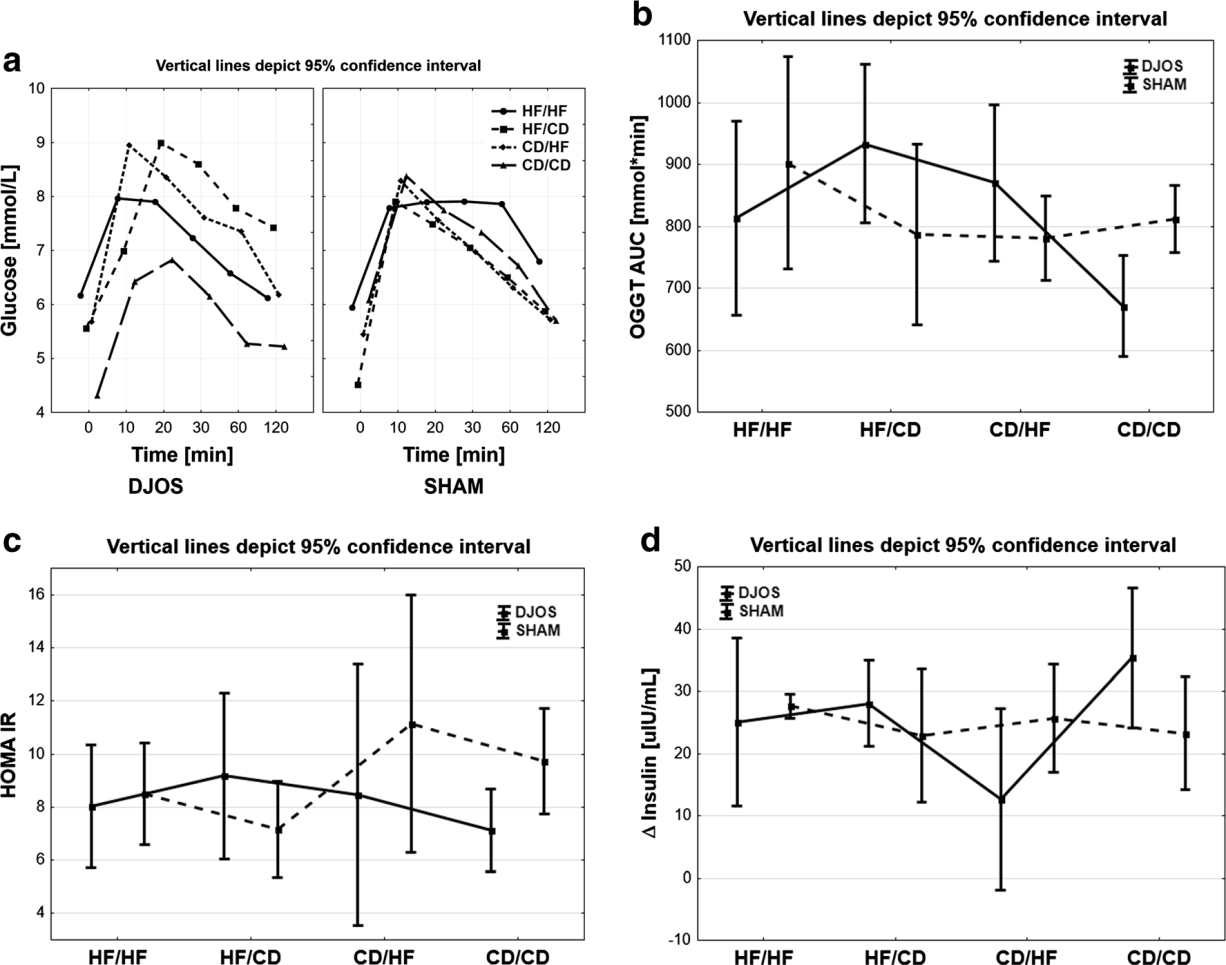
**a** Glucose profiles of OGGT in four groups of diet, according to the operation type. Ninety-five percent confidence interval (95% CI) as calculated from Student’s *t* test. **b** AUC of glucose profiles of OGGT in four groups of diet, according to the operation type. 95% CI as calculated from Student’s *t* test. **c** Mean values of HOMA-IR in four groups of diet, according to the operation type. 95% CI as calculated from Student’s *t* test. **d** Mean values of delta insulin in four groups of diet, according to the operation type. 95% CI as calculated from Student’s *t* test

No changes between the two operation types were observed for groups HF/HF (*p* = 0.499), HF/CD (*p* = 0.073), and CD/HF (*p* = 0.252). A statistically significant difference in time profile course in the group CD/CD was observed (*p* < 0.01). In SHAM-type operations, no statistically significant differences in time profile course were observed. Nevertheless, there was no common glucose pick in the profile of OGTT for the HF/HF group in comparison with other groups. In the DJOS-type operation, differences were observed between groups HF/HF and CD/CD (*p* < 0.05), HF/CD and CD/CD (*p* < 0.001), and CD/CD and CD/HF (*p* < 0.01). The highest glucose tolerance was observed for the CD/CD and HF/HF groups, while a change of diet (HF/CD, or CD/HF) resulted in disabled glucose tolerance (Fig. 2a).

### AUC_OGTT_

No statistically significant changes between the two operation types were observed for the groups HF/HF (*p* = 0.205) and CD/HF (*p* = 0.207). In the HF/CD group, AUC_OGTT_ was statistically significantly higher for DJOS-type surgery than in the SHAM type (*p* < 0.05), while in the CD/CD group, the opposite was found, i.e., the AUC_OGTT_ was higher in the SHAM group compared with the DJOS group (*p* < 0.05). For the DJOS operation groups, the following statistically significant differences were noted: group CD/CD has lower values of AUC_OGTT_ than the HF/HF (*p* < 0.05), HF/CD (*p* < 0.001), and CD/HF groups (*p* < 0.01; Fig. 2b). In contrast, there were no significant changes in the observed values of HOMA-IR between DJOS and SHAM groups (Fig. 2c; Table 1).

**Table 1:** Descriptive statistics and results of two-way analysis of variance

	DJOS				SHAM				*p* ANOVA		
	HF/HF	HF/CD	CD/HF	CD/CD	HF/HF	HF/CD	CD/HF	CD/CD	Group	Op.	Int.
GLP-1 (pg/mL)	43.8 ± 4.4	24.5 ± 4.8	25.0 ± 8.4	28.5 ± 5.0	20.5 ± 6.2	30.4 ± 2.2	36.4 ± 11.8	26.3 ± 8.3	0.338	0.288	*< 0.001*
Eq (kcal)	98.5 ± 22.3	84.1 ± 16.9	148.9 ± 10.9	87.1 ± 15.5	110.3 ± 33.1	80.2 ± 21.6	113.9 ± 24.2	90.8 ± 12.9	0.327	*< 0.001*	*< 0.05*
ΔGIP (pg/mL)	35.8 ± 42.7	26.5 ± 79.0	− 46.8 ± 62.8	− 8.6 ± 60.7	107.2 ± 66.0	45.9 ± 58.9	33.9 ± 79.3	− 79.9 ± 84.4	0.197	*< 0.001*	*< 0.05*
ΔGLP-1 (pmol/L)	13.8 ± 9.1	26.2 ± 8.5	25.3 ± 10.6	36.6 ± 13.9	− 32.3 ± 13.9	14.0 ± 5.34	8.0 ± 9.4	12.8 ± 8.7	*< 0.001*	*< 0.001*	*< 0.001*
ΔINS (uIU/mL)	25.0 ± 14.6	28.0 ± 6.6	12.7 ± 11.8	36.4 ± 10.8	27.7 ± 2.1	22.9 ± 8.7	25.7 ± 8.2	23.2 ± 10.8	0.855	0.104	*< 0.05*
HOMA-IR	8.0 ± 2.5	9.2 ± .0	8.5 ± 4.0	7.1 ± 1.3	8.5 ± 1.8	7.2 ± 1.7	11.1 ± 4.6	9.7 ± 2.2	0.260	0.484	0.163

Mean values ± standard deviation or median (lower–upper quartile). The numbers in italics is the statistical significance (*p*<0.05)

*Op.* operation type. *Int.* interaction between group and operation type

### Insulin

Statistically significant changes between DJOS and SHAM surgeries were observed for the HF/HF (*p* < 0.05) and the CD/HF groups (*p* < 0.05, Table 1). For both DJOS and SHAM surgeries, statistically significant differences between HF/HF and other groups were observed (1 vs. 2, 1 vs. 3, 1 vs. 4; *p* < 0.001, Fig. 2d).

### GLP-1 and ΔGLP-1

**Fig. 3:**
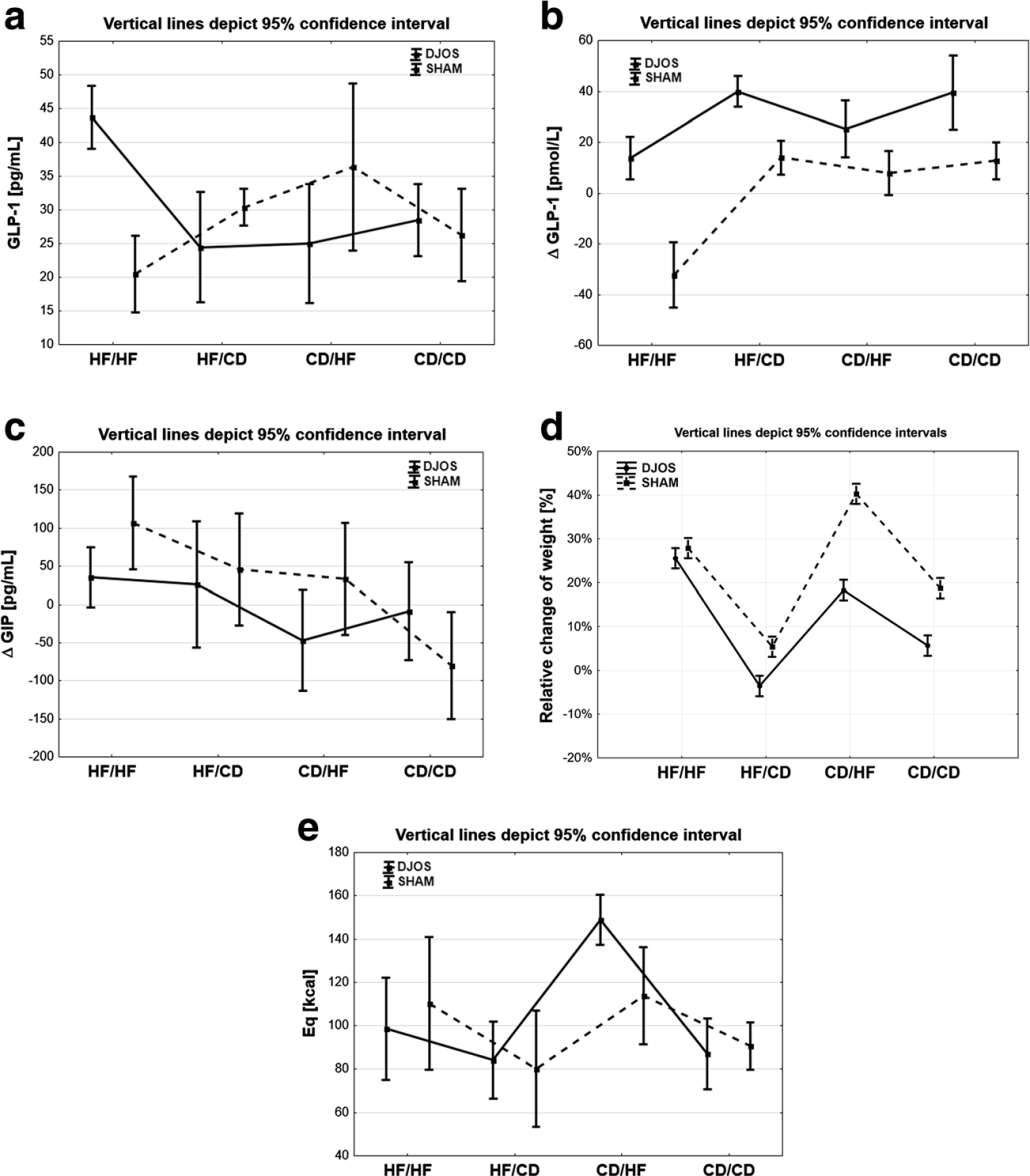
**a** Mean values of glucagon-like peptide 1 in four groups of diet, according to the operation type. 95% CI as calculated from Student’s *t* test. **b** Mean values of delta glucagon-like peptide 1 in four groups of diet, according to the operation type. 95% CI as calculated from Student’s *t* test. **c** Mean values of delta gastric inhibitory polypeptide in four groups of diet, according to the operation type. 95% CI as calculated from Student’s *t* test. **d** Weight profiles in four groups of diet, according to the operation type. 95% CI as calculated from Student’s *t* test. **e** Mean values of Eq in four groups of diet, according to the operation type. 95% CI as calculated from Student’s *t* test

Statistically significant changes between SHAM and DJOS surgeries were observed for all analyzed groups: HF/HF (*p* < 0.001), HF/CD (*p* < 0.05), CD/HF (*p* < 0.01), and CD/CD (*p* = 0.001). In the HF/HF group, ΔGLP-1 was statistically significantly lower for DJOS surgery in comparison with other groups (1 vs. 2, 1 vs. 3, 1 vs. 4; *p* < 0.05, *p* < 0.05, *p* < 0.01). For the same type of surgery, the CD/HF group was statistically significantly lower in comparison with the CD/CD group (*p* < 0.05). In the SHAM surgery group, statistically significant differences between HF/HF and the following groups were observed (1 vs. 2, 1 vs. 3, 1 vs. 4, *p* < 0.001; Fig. 3a, b).

### ΔGIP

Only the CD/HF group (*p* < 0.05) showed statistically significant changes of ΔGIP after SHAM and DJOS surgeries, respectively. After DJOS surgery, ΔGIP was statistically significantly lower in the CD/HF group compared with the HF/HF group (*p* < 0.05). After SHAM surgery, the ΔGIP value was statistically significantly lower for the CD/CD group compared with the other groups (1 vs. 4, 2 vs. 4, 3 vs. 4, *p* < 0.001, *p* < 0.01, *p* < 0.01) and between the HF/HF and CD/HF groups (*p* < 0.05; Fig. 3c).

It can be concluded that statistically significant changes of almost all analyzed parameters were observed in the CD/HF groups following either DJOS or SHAM surgery (Table 2). It suggests that a change of diet, irrespective of surgery, crucially affects all these parameters. The type of surgery was, however, shown to be of fundamental importance for the GLP-1 serum concentration (Fig. 3a, b). The effect of HF on selected parameters like insulin and GLP-1 was clearly visible when comparing DJOS and SHAM surgeries.

**Table 2:** Results of multiple comparisons in contrast analysis

Post hoc	DJOS vs. SHAM				DJOS						SHAM					
	1. HF/HF	2. HF/CD	3. CD/HF	4. CD/CD	1 vs. 2	1 vs. 3	1 vs. 4	2 vs. 3	2 vs. 4	3 vs. 4	1 vs. 2	1 vs. 3	1 vs. 4	2 vs. 3	2 vs. 4	3 vs. 4
INS	*< 0.05*	0.471	*< 0.05*	0.212	*< 0.001*	*< 0.001*	*< 0.001*	0.729	0.069	0.137	*< 0.001*	*< 0.001*	*< 0.001*	0.364	0.150	*< 0.05*
Eq	0.317	0.762	*< 0.01*	0.748	0.238	*< 0.001*	0.351	*< 0.001*	0.801	*< 0.001*	*< 0.05*	0.753	0.078	*< 0.01*	0.381	*< 0.05*
ΔGIP	0.058	0.643	*< 0.05*	0.061	0.809	*< 0.05*	0.252	0.072	0.381	*0.341*	0.135	*< 0.05*	*< 0.001*	0.766	*< 0.01*	*< 0.01*
ΔGLP-1	*< 0.001*	*< 0.05*	*< 0.01*	*< 0.001*	*< 0.05*	*< 0.05*	*< 0.001*	0.880	< 0.05	*< 0.05*	*< 0.001*	*< 0.001*	*< 0.001*	0.830	0.844	0.375
HOMA-IR	*< 0.01*	0.068	*< 0.05*	0.310	0.858	0.639	*< 0.01*	0.510	*< 0.01*	*< 0.001*	0.502	0.437	0.510	0.589	0.938	0.796

### Weight

After surgery, body weight development was largely similar in the DJOS and SHAM groups (Fig. 3d). Statistically significant differences of weight changes were observed for groups HF/HF and HF/CD (*p* < 0.001) at each time of measurement. The overall food intake pattern was similar in the analyzed groups of DJOS and SHAM animals (Fig. 3e). There was a significant change in food intake between the CD/HF group and other the studied groups for DJOS and SHAM. A change of diet to a more caloric one after DJOS surgery did not lead to significant weight loss when the surgery was not combined with gastric restriction. In our experiment, we could observe that a HF reduced the effects of the surgery, in contrast with other reported types of surgery [23].

## Results of Liver Histology

**Fig. 4:**
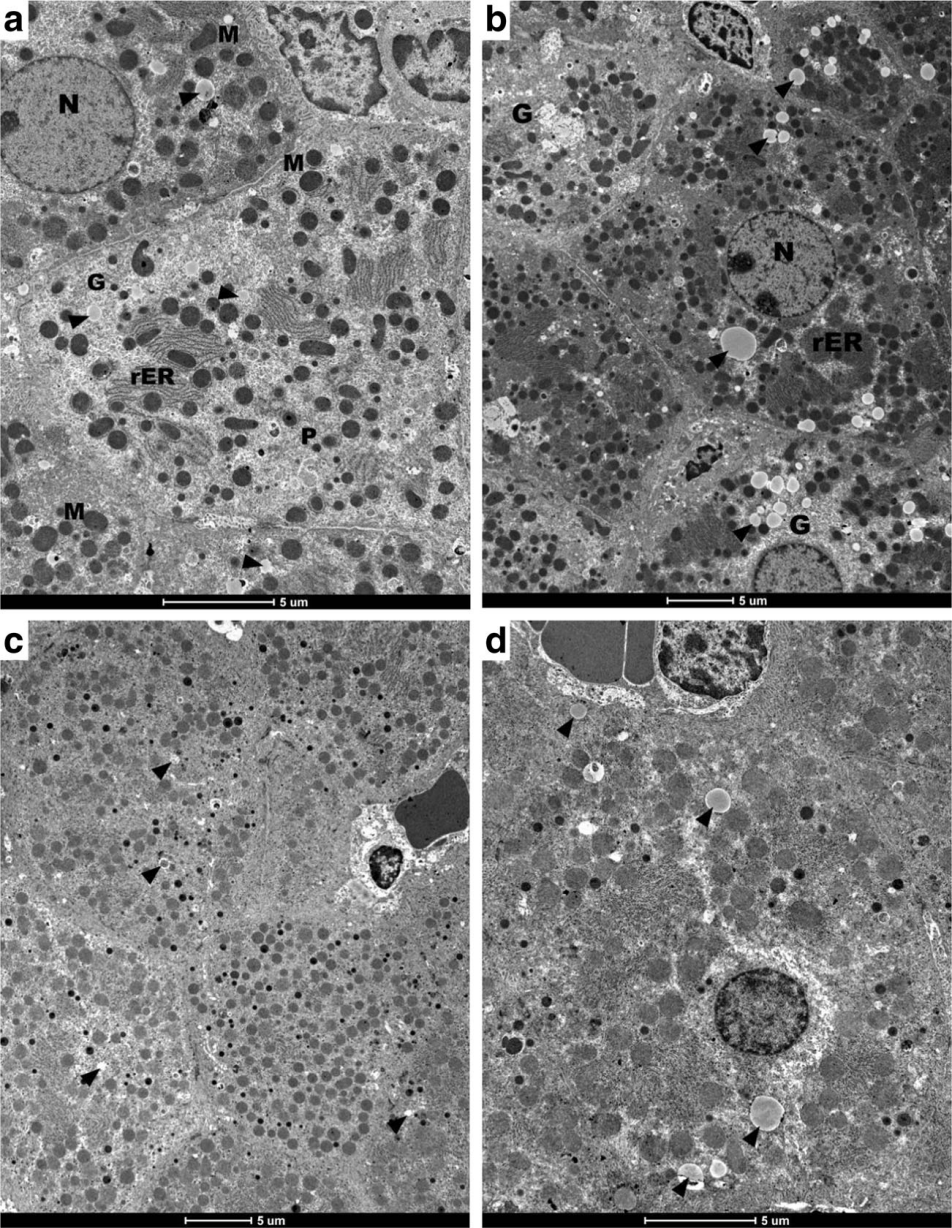
**a**–**d** Electron micrographs of rat liver (HJ/DJOS/HF group) showing hepatocytes with euchromatic nucleus (*N*) of regular outline and prominent nucleolus. The cytoplasm contains round and oval mitochondria (*M*), multiple arrays of rough endoplasmic reticulum (*rER*), glycogen (*G*), multiple peroxisomes (*P*), and a few lipid droplets of different sizes (*arrowheads*) are noticed in cytoplasm

**Fig. 5:**
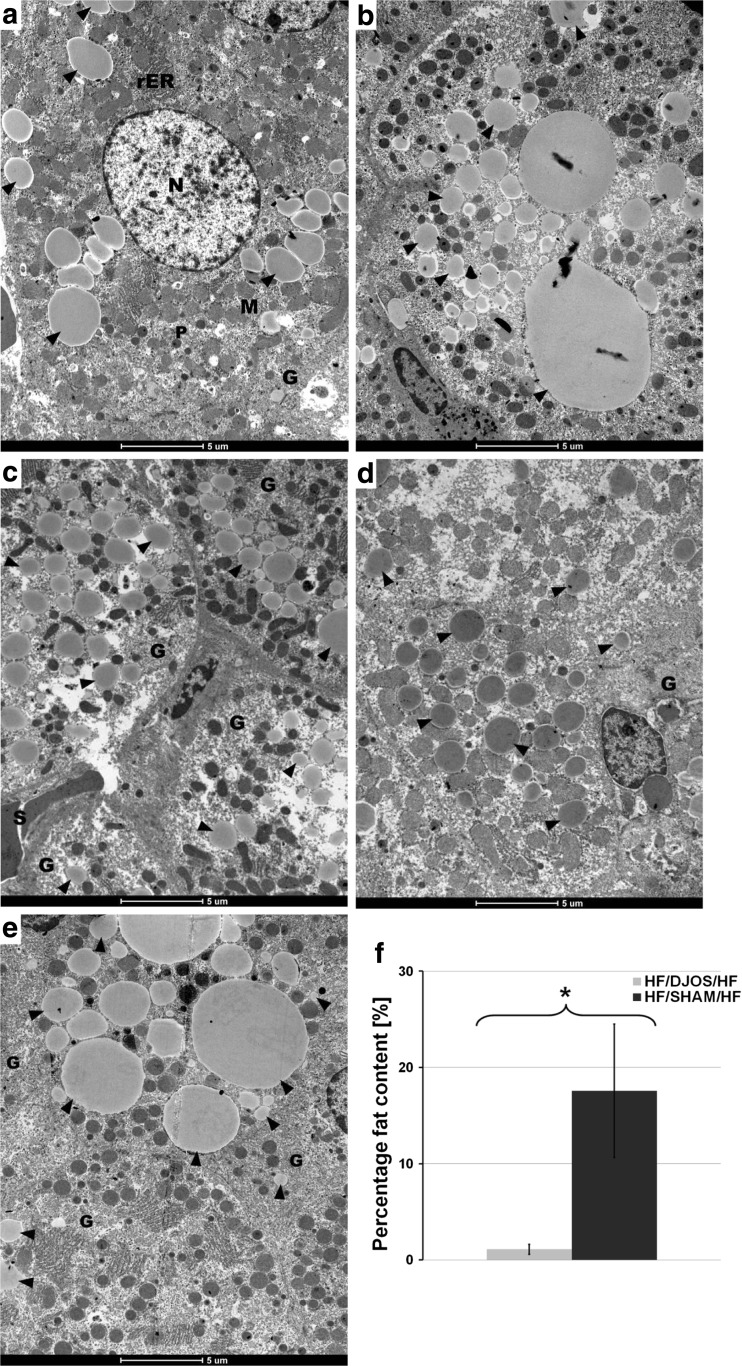
**a**–**f** Electron micrographs of rat liver, HF/SHAM/HF group. **a**–**e** Multiple and different size lipid droplets (*arrowheads*) occupy most of the cytoplasm. Moreover, the cytoplasm contains mitochondria (*M*), rough endoplasmic reticulum (*rER*), and single lysosomes (*L*). **a** Two adjacent hepatocytes enclosing a bile canaliculus, note little amount of condensed heterochromatin within the nucleus (*N*). **c**, **d** The blood sinusoids (*S*) between hepatocytes are visible. **f** Statistical analysis between HJ/DJOS/HF group (*light bar*) vs. HF/SHAM/HF group (*dark bar*). *Asterisk* indicates statistical difference at *p* < 0.05

Histology analysis of liver tissues from the HF/HF groups subjected to DJOS and SHAM were analyzed by transmission electron microscopy (Figs. 4a–d and 5a–f). In order to indirect quantification of fat content, obtained images were analyzed statistically. From each photograph, total surface of the all-fat droplets were calculated with respect to whole visible tissue area. Electron microscopy studies revealed the presence of single small lipid droplets in hepatocytes (Fig. 4a–d). Thus, it can be inferred (1.11 ± 0.52%, Fig. 5f) that no fat accumulation was observed 8 weeks after DJOS surgery in the liver of animals which were kept on a HF diet (Fig. 4a–d). In contrast, fat accumulation visible as numerous lipid droplets of variable sizes in the hepatocytes cytoplasm (17.56 ± 6.94%; Fig. 5f) was observed after SHAM surgery. Furthermore, the hepatic tissues revealed normal ultrastructure in animals of both groups.

## Discussion

It is estimated that up to 90% of people with T2D suffer from obesity, and 80% of subjects with T2D are diagnosed with metabolic syndrome [24]. But it has also been shown that body weight and type 2 diabetes are not in direct causal relationship because 10% of diabetic patients are thin and around 75% of obese patients are not diabetic [10]. Meta-analysis of weight and bariatric surgery shows that the most efficient bariatric procedures, in terms of weight loss, are the biliopancreatic diversion/duodenal switch groups followed by gastric bypass, gastroplasty, laparoscopic adjustable gastric banding, and that the relative effectiveness of these treatments is similar up to about 2 years after the intervention [10]. The treatment of obesity and type 2 diabetes with metabolic surgery influences the physiological role of incretins like GLP-1, GIP, and PYY, which are important players in glucose homeostasis improvement [25]. DJB surgery has also been reported to result in increased GLP-1 secretion [25–28] and is also associated with the improvement of sensitivity to insulin [29].

### Insulin Intolerance and Incretins

Here, it is reported for the first time that a proper diet applied before and after DJOS surgery is crucial for body weight, glucose tolerance, and insulin resistance but not for GLP-1 and hepatic concentration of fat, which depends on the type of surgery one undergoes. GLP-1 plasma concentration, measured before and after surgery, was significantly different for DJOS and SHAM groups despite whichever of the diets was applied. Interestingly, after the surgery, the GLP-1 concentration was significantly higher in the group maintained on a HF when compared with other dietary groups and SHAM animals. It was also associated with higher glucose tolerance and the lowest plasma glucose levels in the group maintained on a HF. In rats, a HF has a negative impact on GLP1-r expression, which is reduced after 3 months of feeding rats, leading to nonalcoholic steatohepatitis (NASH) and nonalcoholic fatty liver disease (NAFLD) [30]. The presence of GLP-1 receptors on rats’ hepatocytes, as well as increased GLP-1 levels after surgery, has a direct effect on hepatocytes by activating the genes involved in fatty acid β-oxidation and insulin sensitivity [30]. In our previous study, we showed that long segment ileal transposition surgery shows a rapid rise in GLP-1 and PYY levels, thus leading to early amelioration of glucose control. In this study, we can conclude that GLP-1 mediates the metabolic benefits of DJOS in a weight loss-independent and diet-independent manner. Reversely, GIP plasma levels, before and after surgery, were lowered in DJOS groups when compared with the SHAM groups, except for those groups maintained only on a CD (before and after the surgery). GIP is a gastrointestinal hormone released in response to nutrient ingestion and potentiate glucose-stimulated insulin secretion. GIP is known to increase the volume of adipose tissue by binding to the GIP receptor located on the adipocytes and by accelerating fat deposition and expansion of fat depots by increasing insulin secretion from pancreatic β-cells [31, 32]. GIP has a physiological role for nutrient uptake into adipocytes and is a key molecule linking over-nutrition to obesity. Excessive fat intake in rats induces GIP gene expression and hypersecretion of GIP. This increases nutrient uptake in the adipocytes leading to obesity and insulin resistance. Reversely, inhibition of the GIP signal in GIP receptor knockout (KO) mice prevents insulin resistance as well as obesity [33]. Yamada has shown that in the situation of GIP receptor aberration, fat is not properly accumulated in adipocytes and is used as the preferred energy source [33]. Our study also shows that exclusion of duodenum and the proximal part of jejunum leads to a decrease of GIP levels after surgery but with an unexpected exception for the group maintained on a HF only. Over-nutrition with fat is a very strong factor, which dramatically reduces the effects of DJOS surgery. Nevertheless, comparing the changes in ΔGIP levels, the effect of DJOS was visible in animals maintained on a HF or mixed CD/HF and HF/CD diets, with the strongest effect occurring in the CD/HF group. The change of the diet from a CD to a HF had the strongest impact on GIP levels, which were significantly increased in the SHAM animals when compared with animals that underwent the DJOS surgery. Many studies show positive results of bariatric surgery on the resolution of DM2T and obesity [25, 26, 34]. In summary, we have shown that GLP-1 levels increase following DJOS irrespective of body weight changes. Transition to another diet after surgery impaired insulin tolerance and GIP effect more than keeping the animals on the same type of diet. After OGTT, animals in DJOS showed improved glucose tolerance for HF/HF and CD/CD in relation to the SHAM animals. DJOS rats showed significantly reduced AUC_OGTT_ for the HF/HF and CD/CD groups.

### Diet Effect

Previous experimental model studies have shown a recurrence of glucose intolerance after an initial improvement directly after surgery [35]. Our results show that different types of diets, applied before and after surgery, may be considered a valuable and close to human behavior model for the investigation of the mechanism of glucose intolerance after bariatric surgery. In this study, we decided to use a HF in order to induce obesity and insulin resistance. A HF is considered a factor which decreases insulin ability for suppression of hepatic glucose production and lowers glucose uptake [36]. After 8 weeks on a HF, we observed an impaired insulin tolerance in all animals selected for DJOS surgery. After DJOS surgery, the glucose tolerance was ameliorated in both the HF/HF and CD/CD groups but not in the groups of animals whose diet was changed from a HF to a CD and from a CD to a HF. The glucose amelioration in HF/HF subjects was found to be comparable with the control group, which can be an effect of ketogenic properties of a HF. This contrasts with other findings, which showed that a high-caloric diet was an important reason for the retreat of improvement in diabetes after surgery [35]. The amount of food and quality of food was not reduced and changed before and after surgery. We appreciate that for some of the studied parameters, HF diet had stronger impact than surgery itself. This effect would probably be much more accentuated if connected to gastric restriction. We should stress here that time after surgery is also an important or even crucial influential factor, so to compare our results with the others, the similar protocols are necessary, meanwhile the available results present the full spectrum of surgeries, diets, and periods.

### Impact of DJOS on Body Weight

In this study, DJOS surgery was shown to have very little impact on body weight reduction. In all groups, the body mass was increased after DJOS surgery. In the DJOS groups, the body weight had no negative effect on insulin levels, glucose tolerance, or liver fat deposition. We can conclude that after DJOS, even though the body weight increased, the amelioration of glucose tolerance was reached for the HF/HF and CD/CD groups but not for the mixed diet groups (HF/CD and CD/HF). Changes in diet after surgery influenced the glucose-stimulated insulin secretion. HF diet, less carbohydrates, and a more obesogenic diet applied before and after the surgery decreased levels of plasma insulin when compared with other groups. It was combined with a continuous decrease in plasma glucose level in the HF/HF group but not in the CD/HF group, which might be associated with a high level of GLP-1 [27, 29]. Liu and coworkers observed that this trend may change with time. After long-term observation, 16 weeks after DJB surgery, the increased caloric intake reduced the beneficial effects of the surgery and resulted in decreased insulin sensitivity, thus leading to re-impairment of glucose tolerance and reversal of the initial improvement [35]. Our results show that DJOS surgery did not reduce body weight in groups maintained on a HF, but instead prevented lipid accumulation in the liver. Similar results were obtained for the remediation of NAFLD and NASH after Roux-en-Y gastric bypass (RYGB) and sleeve gastrectomy surgeries [37]. We note that the gastric size was not reduced after DJOS. According to current literature, we may however conjecture that gastric reduction will influence body mass and amount of food consumed by studied animals. That was the main reason why we decided to study DJOS and ketogenic diet without any supplementary surgery. In that aspect, we are here able to study effects of DJOS on selected parameters not influenced by changed, limited amount of food consumed, or other effects of gastric reductions.

## Conclusions

In this study, the effect of DJOS surgery was analyzed after 8 weeks with respect to body weight, glucose tolerance, and incretins in Sprague-Dawley obese rats maintained on an obesogenic diet, which is known to produce metabolic dysfunction in the liver and on a regular controlled diet. The main findings are (i) in short-term DJOS surgery, it was found not to reduce body weight in the studied groups, except the control group CD/CD where a slight reduction was observed, (ii) selected incretin GLP-1 levels increased following DJOS irrespective of body weight change, (iii) DJOS surgery prevents lipid accumulation in the liver for the HF/HF groups, (iv) change of diet after surgery impaired insulin tolerance and GIP effect more than keeping the animals on the same type of diet before and after surgery, and (v) based on OGTT, animals which underwent DJOS surgery showed improved glucose tolerance for HF/HF and CD/CD. For those groups, the 30- and 60-min plasma glucose levels were consistently lower in relation to SHAM animals, (vi) DJOS animals exhibited significantly reduced AUC_OGTT_ for the HF/HF and CD/CD groups, and therefore ameliorated glucose tolerance, and (vii) DJOS surgery together with HF influenced the level of insulin in all subjects.
